# Crossbreeding of transgenic flax plants overproducing flavonoids and glucosyltransferase results in progeny with improved antifungal and antioxidative properties

**DOI:** 10.1007/s11032-014-0149-5

**Published:** 2014-08-21

**Authors:** Justyna Mierziak, Wioleta Wojtasik, Kamil Kostyn, Tadeusz Czuj, Jan Szopa, Anna Kulma

**Affiliations:** Faculty of Biotechnology, Wroclaw University, Przybyszewskiego 63/77, 51-148 Wrocław, Poland

**Keywords:** Flax, Flavonoids, Fusarium, Cross breeding, Antioxidants, Antifungal activity

## Abstract

Flavonoids are a large group of secondary plant metabolites with many important functions; they play a role in fruit, flower and seed pigmentation and are involved in multiple protective mechanisms. They are very active natural antioxidants, acting as antimicrobial compounds in defense against pathogens, and they protect the plant against various stress factors, including excessive solar radiation and temperature. They are also an animal deterrent. Flax is already a very useful crop plant with nutritional and biomedical applications. With increased phenylpropanoid content, flax plants could be used in the production of improved dietary supplements and antimicrobial agents. The main aim of this study was to engineer a flax variety with increased flavonoid content by crossing two transgenic flax varieties that have heightened flavonoid levels. A mother plant that over expresses genes encoding the flavonoid biosynthesis pathway enzymes chalcone synthase, chalcone isomerase and dihydroflavonol reductase was crossed with plants overexpressing the glucosyltransferase (GT) gene. It was expected that the progeny would display better properties thanks to the simultaneous increases in flavonoid synthesis and stability. In comparison to the control and parental plants, plants of the selected flax lines were found to have increased contents of flavonoids and other phenylpropanoids, including phenolic acids, in their stems and seeds. A significant increase in the secoisolariciresinol diglucoside content was found in the seeds. The antioxidative properties of extracts from W92 × GT crossbreed plants were higher than the control (non-transgenic) and parental plants. These results correlated with the increase in the susceptibility of the crossbreeds to *Fusarium* infection. The increased flavonoid content did not cause any negative phenotypic changes or reduce the yield of seeds.

**Electronic supplementary material:**

The online version of this article (doi:10.1007/s11032-014-0149-5) contains supplementary material, which is available to authorized users.

## Introduction

By using genetic engineering methods to introduce specific modifications to the plant genome, it is currently possible to produce plants with defined properties that have particular potential for industrial, agricultural and medical applications. One line of research focuses on generating plants with a better response to negative environmental influences, including pathogen infection. Part of the plant defense response against pathogens is the production of various secondary metabolites, including flavonoids. These compounds are derivatives of 2-phenyl-γ-benzopirone, and they share a common structure of a carbon skeleton based on flavan (C6–C3–C6) (Pietta 2000).

Flavonoids have diverse chemical structures and multiple important functions in plants. They participate in protection against harmful biotic and abiotic factors, including excessive solar radiation and temperature, mainly by scavenging free radicals. They facilitate the regulation of osmotic pressure during periods of drought or low temperature. They play a role as antimicrobial compounds in plant defense against pathogens, and they aid in preventing the plants from being eaten by herbivores. Flavonoids are also pigments found in fruits, flowers and seeds (Bohm 1998; Joung et al. 2003; Kurt and Evans 1998; Michalik 2010). As natural antioxidants, they also have value for human health (Rice-Evans et al. 1996).

The flavonoid content in plants can be increased through overexpression of a single or multiple genes engaged in flavonoid biosynthesis and/or by increasing their stability. The aim of this study was to increase the flavonoid content in a crossbred flax plant. Flax seeds have a high nutritional value thanks to their high content of polyunsaturated fatty acids, phytosterols, vitamin E, carotenoids, and lignans. The value of the fibers is in their content of antioxidative compounds, making them better than cotton fibers, which only contain cellulose. The presence of natural antioxidants means they are advantageous for the production of wound dressings and surgical threads (Czemplik and Szopa 2009).

Multiple anthocyanins (including pelargonidin, cyanidin and delphinidin derivatives) that play a role in flower pigmentation (Dubois and Harborne 1975) were found in flax tissues. The flavonols 3,7-*O*-dimethoxy herbacetin, kaempferol-3,7-*O*-diglucopyranoside and herbacetin 3,7-*O*-diglucopyranoside were identified in flax seedcakes (Mitek and Gasik 2007). Kaempferol, kaempferol-3-*O*-galactoside, rutin and genistein were found in flax flowers (Stosic et al. 1989). Moreover, various flavonoid C-glucosides and di-C-glucosides were found in flax (Dubois and Mabry 1971; Stosic et al. 1989), including orientin and isoorientin and their derivatives (Wagner et al. 1972; Volk and Sinn 1968).

In our laboratory, we generated a transgenic flax line (W92) characterized by increased flavonoid content arising from simultaneous overexpression of genes coding for three enzymes of the flavonoid biosynthesis pathway: chalcone synthase (CHS), chalcone isomerase (CHI) and dihydroflavonol reductase (DFR). The transgenic flax plants showed increased phenolic acid contents, and, as a consequence, an altered fatty acid content in the seeds and a significant increase in antioxidant capacity. There is evidence that the increased antioxidative properties of the seeds were accompanied by an elevated lignan level. This increase in the antioxidative properties manifested itself as a higher level of plant resistance towards pathogen infection (Lorenc-Kukuła et al. 2005).

Flavonoid glycosylation increases their stability, and it is suggested that accumulating larger amounts of stable flavonoid glycosides in transgenic plants would improve their antioxidative properties. Therefore, our second transgenic flax line overexpressed the glucosyltransferase (GT) gene, which encodes 5-*O*-GT. This enzyme has broad specificity and catalyzes the flavonoid glycosylation reaction (Aksamit-Stachurska et al. 2008). Transgenic GT flax seeds showed increased levels of phenolic acids, anthocyanins, unsaturated fatty acids and secoisolariciresinol diglucoside (SDG). Overexpression of the GT gene caused a significant increase in plant resistance to infections (Lorenc-Kukuła et al. 2009).

The main goal of this study was to obtain plants with increased contents of more stable flavonoids by crossing the transgenic flax lines W92 and GT. The resulting plants were analyzed to determine whether they have even better antioxidative properties than the parental lines and whether such crossbreeding negatively influences the features of the new plants. To the best of our knowledge, this is the first report on transgene stacking in flax through a crossing of transgenic varieties.

## Materials and methods

### Parental and control plants

#### Maternal plants: W92 flax with overexpression of genes participating in flavonoid synthesis

To generate plants that overexpress genes participating in flavonoid synthesis, a multigene construct containing three genes, CHS, CHI and DFR, was prepared. The construct in a binary vector was introduced to the cells of oil flax var. *linola* using *Agrobacterium tumefaciens*. The three-gene construct contained three cDNAs from *Petunia hybrida* encoding the *CHS* gene (EMBL/GenBank number X04080) of 1,380 bp, *CHI* gene (EMBL/GenBank number X14589) of 826 bp and *DFR* gene (EMBL/GenBank number X15537) of 1,330 bp. The cDNAs were cloned into the pBinAR vector in the sense orientation under the control of a strong, non-specific CaMV35S promoter and the octopine terminator (OCS). A modification of the original pBin binary vector, the pBin W88 vector, was used. Additionally, the vector used contained the kanamycin resistance gene (*Kanr*) (Lorenc-Kukula et al. 2005).

#### Paternal plants: GT flax with overexpression of the glucosyltransferase gene

To generate plants that overexpress the GT gene, we used a gene construct consisting of a 1,617-bp fragment of *Solanum sogarandinum* cDNA (EMBL/GenBank number AY033489) under the control of seed-specific napin promoter (NAP) and OCS. The construct was cloned into the pBinAR/NAP vector in the *Bam*HI/*Sal*I site in the sense orientation and introduced to the cells of oil flax var. *linola* using *A. tumefaciens*. The vector additionally contained the kanamycin resistance gene (Lorenc-Kukula et al. 2009).

#### Control plants

The control plants were oil flax var. *linola* (LIN).

### Research material and cultivation conditions

The research material was from the F2 generation of plants which resulted from the crossbreeding of W92 and GT transgenic plants. The crossing was performed in the experimental field by pollination of the pistils of W92 plants (maternal line) with pollen from GT plants (paternal line). The F1 and F2 generations were cultivated in pots under greenhouse conditions with a substratum of peat soil (pH 7.0) and sand (2:1) at 22 °C for 16 h and 15 °C for 8 h with a luminosity of 103 mmol s^−1^ m^−2^ (50 %) and 250 mmol s^−1^ m^−2^ (100 %) and daily watering. Tissue from 4-week-old F2 plants served for the selection of the crossed plants and for the assessment of flavonoid and total phenolic compound contents in the green tissue. The stems and seed capsules of F2 plants were collected 6 months after the planting of those seeds and used for further assays. The analyzed traits of the resultant crossbreeds were compared to the features of the parental plants and the control, non-transgenic plants.

### Selection of transgenic crossbreed plants using PCR

The transgenic plants were selected using PCR with a DNA matrix. For the amplification of the exogenous 679-bp GT (5UGT) gene fragment, the primers were: forward—TGAAGTGTAGCTCAAATGATCC, reverse—CACAGGTACACCTGATGACAG. For the 488-bp exogenous dihydroflavone reductase (DFR) gene, the primers were: forward—CCTACATTTCCCCCTAGTTTA, reverse—CTTTGCCACTTGCATAGTTTTGA. The reaction products were separated by electrophoresis in 1.5 % agarose gel. The gel was stained with ethidium bromide and observed under a UV lamp.

### Flavonoid content assessment of green tissue

Aliquots of 35 mg of the green tissue ground in a mortar were used for the assay. The samples were extracted with 400 µl of methanol and then sonicated in an ultrasound bath for 30 min. Next, the samples were centrifuged at 14,000×*g* for 10 min. The supernatant was collected, dissolved with 400 µl of 1 % 2,4-dinitrophenylhydrazine (DNPH) and incubated at 50 °C for 50 min. Subsequently, 1 ml of 1 % KOH in 70 % ethanol was added to each sample, followed by centrifuging at 1,000 *g* for 15 min. After the centrifugation, the supernatant was transferred to new Eppendorf tubes. The absorbance of the samples was measured at *λ* = 495 nm. The results are presented as equivalents of naringenin, and served for calibration curve preparation (Chang et al. 2002).

### Total phenolic content assessment of green tissue using the Folin–Ciocalteu method

Ground, dry green tissue samples (15 mg) were used for the assay. Extraction was done with 200 µl of methanol/HCl mixture (95:5, v/v). The samples were sonicated for 30 min in an ultrasound bath. After the sonication, the samples were centrifuged at 14,000×*g* for 10 min, and then 1 ml of Folin–Ciocalteu reagent diluted ten times with water was added to the supernatant. After subsequent incubation for 5 min at room temperature, the samples were supplemented with 1.5 ml of 6 % Na_2_CO_3_, and then incubated for 1.5 h at room temperature. Absorbance at *λ* = 725 nm was measured after the incubation. The results obtained are presented as equivalents of gallic acid (Prior et al. 2005).

### Secoisolariciresinol diglucoside (SDG) and phenolic acid content assessment of flax seeds

Five seeds were placed in tubes in three replications. The seeds were ground and defatted with hexane. The defatted seeds were extracted with 0.5 ml of 80 % methanol (v/v) followed by sonication at 70 °C for 15 min, three times. The extracts were centrifuged at 14,000×*g* for 10 min. The supernatant was collected and dried in a vacuum centrifuge. After drying, 0.5 ml of NaOH was added to the samples for hydrolysis. The alkaline hydrolysis was conducted for 48 h at 37 °C. The pH of the extracts was adjusted to 7.

The compounds were assayed using ultra-high-performance liquid chromatography (UPLC) with a 2996 PDA photodiode detector (Waters Acquity UPLC system). The separation was done on a BEH C18 2.1 × 100 mm, 1.7 µm column (Waters Acquity UPLC system) with 0.1 % formic acid (solution A) and acetonitrile (solution B) as the eluent. The spectra were recorded in the range of 200–500 nm. Detection of SDG and other compounds contained in the seeds was performed at *λ* = 280 nm. The separation was done at a flow ratio of 4 ml/min with a gradient flow of 1 min—90 % A and 10 % B, 2–10 min—gradient to 80 % A and 20 % B, 10–12 min—gradient to 0 % A and 100 % B, and 12–13 min—gradient to 90 % A and 10 % B. The compounds were identified based on the retention times and absorbance and MS spectra. The quantities of phenolic acid aglycones and SDG were calculated in comparison to the standards (Sigma-Aldrich, USA) (Żuk et al. 2011). Phenolic acid glucoside concentrations were calculated based on respective aglycones, based on the molar extinction coefficient for those compounds.

### Phenylpropanoid compound content assessment of flax stems

Samples of ground, dry stems (0.25 g) were used in the assay. The samples were extracted with 7 ml of methanol and centrifuged (10 min, 1,750×*g*) three times. The supernatants obtained were combined and dried in a vacuum evaporator, and then suspended in 1 ml of methanol. To assess the content of the cell-wall-bound compounds, the sediments remaining after the methanol extraction were supplemented with 7 ml of 2 M NaOH solution and hydrolyzed for 48 h at 37 °C. After the alkaline hydrolysis, the samples were centrifuged (10 min, 1,750×*g*) and the supernatants were collected. The pH was adjusted to 3.0 and extraction with 7 ml of ethyl acetate was performed three times. Subsequently, the ethyl acetate was evaporated in a vacuum evaporator, and the remainder was re-suspended in 0.4 ml of methanol. The initial methanol extracts and the extracts after alkaline hydrolysis were analyzed by UPLC using a 2996 PDA photodiode detector (Waters Acquity UPLC system). The separation was done on a BEH C18 2.1 × 100 mm, 1.7 µm column (Waters Acquity UPLC system) with 0.1 % formic acid (solution A) and acetonitrile (solution B) as the eluent. The separation was done at 4 ml/min flow ratio with gradient flow: 1 min—95 % A and 5 % B, 2–12 min—gradient to 70 % A and 30 % B, 12–15 min—gradient to 0 % A and 100 % B, and 15–17 min—gradient to 95 % A and 5 % B. The compounds were identified based on the retention times and absorbance spectra, and their quantities were calculated in comparison to the standards (Sigma-Aldrich, USA). The detection was done at *λ* = 280 nm (Wojtasik et al. 2013).

### Lignin content assessment of flax stem cell wall

Total lignin content was measured with the modified ‘acetyl-bromide’ method (Hatfield et al. 1999). In brief, water was poured onto dried and finely ground samples (10 mg) and heated for 1 h at 65 °C, and further filtered through GF/A filters, which was followed by washing several times with different organic solvents (in turn: ethanol, acetone, diethyl ether). The pellets obtained were dried for 12 h and then, after adding 25 % acetyl-bromide in acetic acid, the samples were incubated for 2 h at 50 °C and further dissolved in 10 ml of 2 N NaOH mixed with 12 ml of acetic acid. After 12 h of incubation at room temperature the lignin content was measured spectrophotometrically at 280 nm. The results were given as an equivalent of coniferyl alcohol.

### Total pectin content assessment of flax stem cell wall

Total pectin content was determined using the biphenyl method (Blumenkrantz and Asboe-Hansen 1973) after prior hydrolysis with concentrated sulphuric acid (Ahmed and Labavitch 1978). 10-mg samples of ground, dry stem tissue were used for pectin content determination.

To remove contamination the samples were treated with 96 % ethanol at 100 °C, 80 % ethanol at 80 °C, chloroform:methanol solution (1:1 v/v) at 40 °C and then acetone at room temperature. After drying, the samples were hydrolyzed with concentrated H_2_SO_4_ in an ice bath. After being diluted with water and centrifuged, the supernatant containing pectin was collected in new tubes and supplemented with 4 M sulfamic acid potassium sulfonate solution, pH 1.6, Na_2_B_4_O_7_ in H_2_SO_4_, then incubated for 20 min at 100 °C. Finally, *m*-hydroxybiphenyl was added to measure absorption at 525 nm. The results were given as an equivalent of glucuronic acid.

### Cellulose content assessment of flax stem cell wall

Cellulose content in the cell wall was assayed using the anthrone method (Ververis et al. 2004). 15-mg samples of ground, dry stem tissue were used for the assay.

The samples were incubated for 1 h at 100 °C in a mixture of nitric and acetic acid (1:8 v/v). The samples were centrifuged, the supernatant discarded, and the pellets washed twice with distilled water, then dissolved in 67 % H_2_SO_4_ (v/v) and incubated for 1 h. The samples were diluted ten times and cooled anthrone reagent was added. The cellulose content was measured spectrophotometrically at 620 nm.

### Fatty acid content assessment of flax seeds

#### Esterification of fatty acids

Fatty acids were measured according to Lorenc-Kukuła et al. (2005) and the modified international standard ISO method (ISO 12966-2 2011). Ground flax seeds were placed in glass tubes with Teflon-sealed screw caps and supplemented with 500 µg of pentadecanoic acid as an internal standard. Each sample was prepared in triplicate. 1 ml of 0.5 M KOH in anhydrous methanol was added to each sample. The samples were then shaken well and incubated for 30 min at 70 °C. After cooling, 1 ml of 1.25 M HCl in anhydrous methanol was added and each sample was again mixed and incubated at 70 °C for 30 min. The samples were cooled and 1 ml of hexane and 3 ml of saturated NaCl were added to each. The samples were then mixed well for 5 min and the hexane layers were collected. Fatty acid methyl ester (FAME) extraction was repeated by adding 1 ml of hexane, and the collected hexane was stored in 4 °C until measurement (until the next day at the latest).

#### FAME analysis

FAME analysis was done using an Agilent 7890A gas chromatograph with an FID detector equipped with a capillary column for the determination of fatty acids DB-23 (60 m, 0.25 mm, 0.25 µm). Before the analysis, a series of FAME standards (37 FAME Standard Mix from Supelco and RM-2 Flax Oil FAME Standard Mix from Sigma-Aldrich) were run to determine the correct retention times for each FAME. Each sample was placed in a glass autosampler vial and run in the same conditions as the standard mixtures. Each FAME was identified by retention time and its quantity was calculated according to the internal standard. The esterification and measurements were done in triplicate.

### Determination of antioxidative potential of methanol extract of flax green tissue using DPPH and FRAP methods

Samples of green tissue (100 mg) from flax tissue cultures were ground after freezing in liquid nitrogen. The samples were extracted three times with 0.5 ml methanol, centrifuged each time (10 min, 18,000×*g*) and the supernatants were collected and used for the DPPH and FRAP analyses of the antioxidative properties.

#### DPPH method

The 6-µl aliquots of the plant extracts studied were added to 200 µl of 0.1 mM DPPH reagent (1,1-diphenyl-2-picrylhydrazyl) and incubated for 15 min in darkness at room temperature, and absorbance was then measured at *λ* = 515 nm. The control sample was 200 µl of DPPH and 6 µl of methanol. The blank sample was pure methanol. The antioxidative properties were expressed as antioxidative potential (equal to the inhibition of the free-radical reaction expressed as a percentage) according to the following equation:

$$P = 1 - \frac{{{\text{Abs}}_{\Pr } }}{{{\text{Abs}}_{\text{C}} }} \times 100\,\% ,$$

where *P* is the antioxidative potential, Abs_Pr_ is the absorbance of the examined sample solution and Abs_c_ is the absorbance of the DPPH radical solution (Williams et al. 1995).

#### FRAP method

Samples of the studied and control plant extracts (6 µl) were added to 200 µl of FRAP reagent (300 mM acetate buffer at pH 3.6, 20 mM FeCl_3_, 10 mM 2,4,6-tripyridyl-*s*-triazine in a 10:1:1 ratio) and incubated at 37 °C for 4 min. The absorbance was measured at *λ* = 595 nm. The antioxidative properties were expressed as antioxidative potential equal to the difference between absorptions of the sample before and after the 4-min incubation (Benzie and Strain 1996).

### Assessment of tissue culture plant resistance to *Fusarium culmorum* and *F. oxysporum*

*Fusarium culmorum* and *F. oxysporum* were a kind gift from the Plant Breeding and Acclimatization Institute (Poznan, Poland). Five-day-old inoculums of *F. culmorum* and *F. oxysporum* with areas of approximately 0.8 cm^2^ were transferred to 10 ml of potato dextrose agar (PDA) medium in 50-ml tubes and cultured for 1 day at 28 °C. Discs of agar-solidified plant medium (4.4 g/l Murashige and Skoog medium, 4 g/l sucrose, 5 g/l agar) with volumes of 10 cm^3^ and heights of 2 cm were then placed in the tubes. Four-week-old tissue culture plants without roots were placed on the discs. The tubes were moved to a greenhouse and observed daily. The plants exposed to *F. culmorum* were analyzed after 3 days, and those exposed to *F. oxysporum* after 7 days.

### Statistical analysis

The results are presented as the means of three independent replications ± standard deviations. Variance analysis (ANOVA) was performed using the Tukey test. Statistica 9 (Statsoft) software was used for the statistical analysis.

## Results

### Selection of the transgenic crossbred plants using PCR

The plants resulting from the crossbreeding of plants of the transgenic lines W92 (overexpressing three genes of the flavonoid synthesis pathway) and GT (overexpressing GT), designated W92 × GT, were selected using PCR (Fig. S1). After 50 crossing events we obtained 44 seed pods with the average number of seeds per pod ranging from 3 to 9. After seeding, only 147 seedlings grew, of which c. 62 % were bearing parental genes. From them, 13 that contained both exogenous genes were chosen because they also had the highest flavonoid contents, as measured spectrophotometrically (data not shown). The seeds of the selected transgenic plants were planted in pots and cultivated in the greenhouse to obtain the next generation (F2). The F2 generation plants were screened analogously to confirm the transgenicity and stability of the transgene. Seven of the 13 plant lines were chosen for further analyses. These seven were all characterized by the highest flavonoid contents, as measured spectrophotometrically (data not shown). They were designated numerically: 2.9, 15.1, 15.2, 17.5, 37.2, 38.3 and 39.6.

### Phenotypic analysis of W92 × GT plants

The W92 × GT crossbreeds grown in the greenhouse did not differ from the control plants in terms of overall shape and size, or color and size of leaves and flower petals. All the seeds obtained were yellow. The mean mass of the W92 × GT seeds was about 24 % higher than that of the seeds from the control plants (LIN), but about 29 % lower than the mean mass of seeds from the parental plants. The average number of seeds per capsule from the crossbreeds was higher than for the control and parental plants. The contents of the main polymers of the cell wall (cellulose, pectin and lignin) were not different from those for the control and parental plants (assayed in the stem tissue, Supplementary Fig. S2).

### Assessment of flavonoid and total phenolic compound contents of flax green tissue

**Fig. 1:**
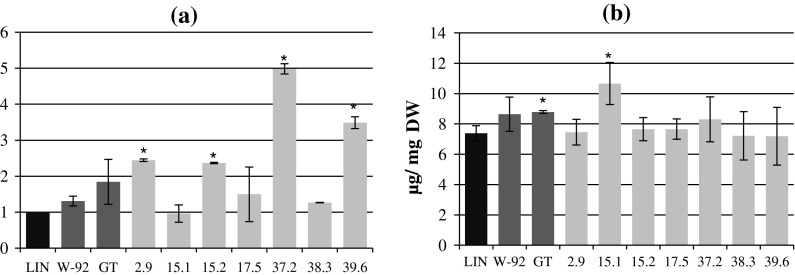
**a** Analysis of the flavonoid levels in the green parts of the W92 × GT, control (LIN) and parental (W92, GT) plants. The procedure is described in the “Materials and methods” section. The results are the mean values ± SD of three independent experiments (*n* = 3, *p* < 0.05) and are presented in relation to the values obtained for the control plants (where the values were 1). **b** The content of phenolic compounds in the green parts of the W92 × GT, control (LIN) and parental (W92, GT) plants. The assay was performed using the Folin–Ciocalteu method. The procedure is described in the “Materials and methods” section. The results are the mean values ± SD of three independent experiments (*n* = 3). *Statistically significant difference compared to LIN at *p* < 0.05. *DW* dry weight. **a** Flavonoids, **b** total phenolic compounds

Lines 37.2 and 39.6 were characterized with the highest flavonoid contents in their green tissue: respectively 398 and 248 % compared to the control plants, 280 and 166 % compared to the maternal plants, and 170 and 89 % compared to the paternal plants. Three W92 × GT plant lines (15.1, 17.5 and 38.3) had similar flavonoid contents to the paternal plants. The data are presented in Fig. 1a.

The total phenolic content in the green tissue of the W92 × GT plants differed from that of the control plants (LIN) and parental plants (Fig. 1b). Only 15.1 showed a statistically significant difference compared to the control (LIN).

### UPLC analysis of phenolic compounds in flax stems

**Fig. 2:**
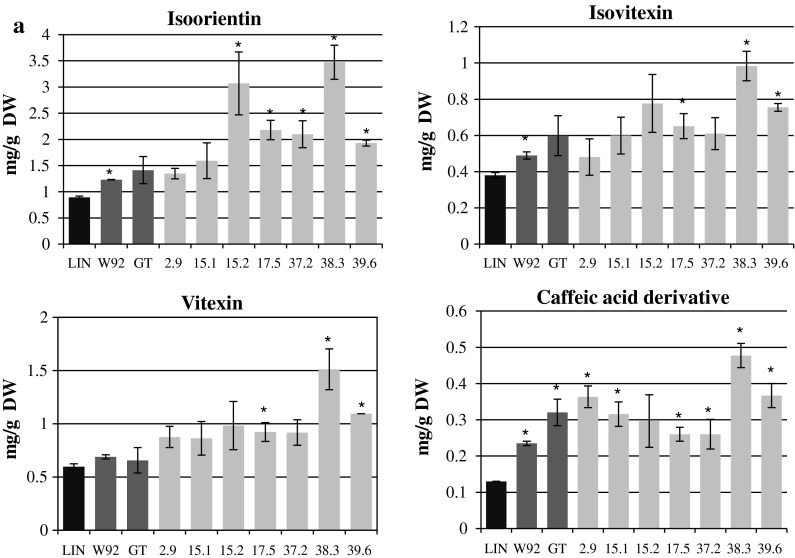

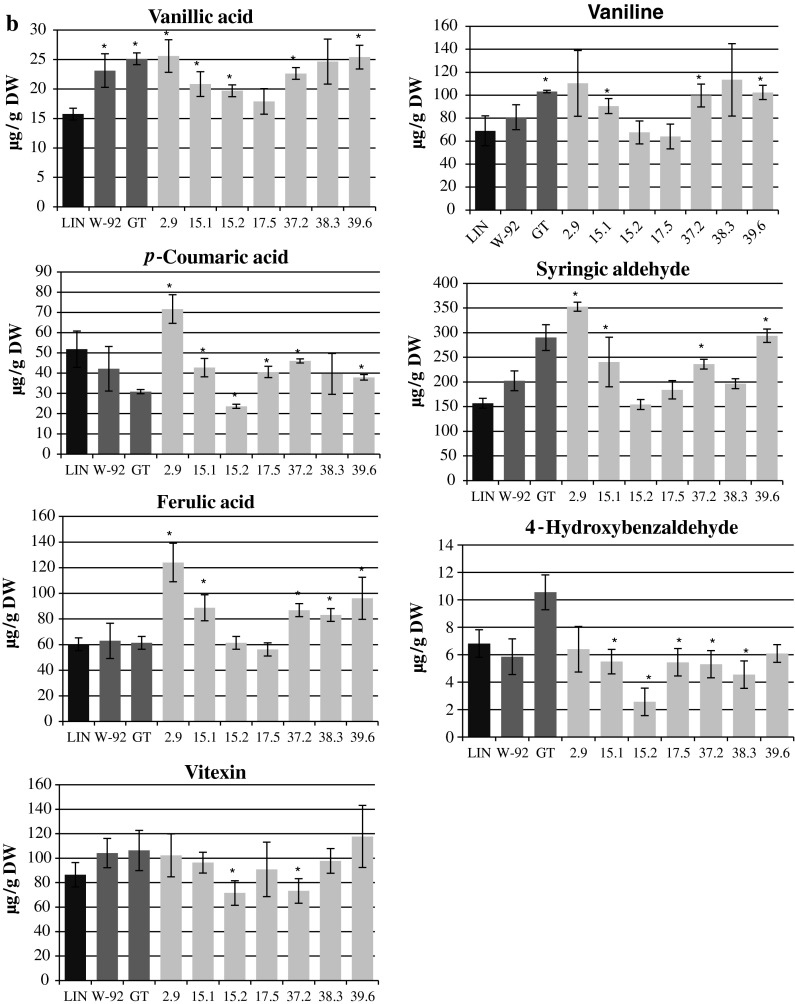

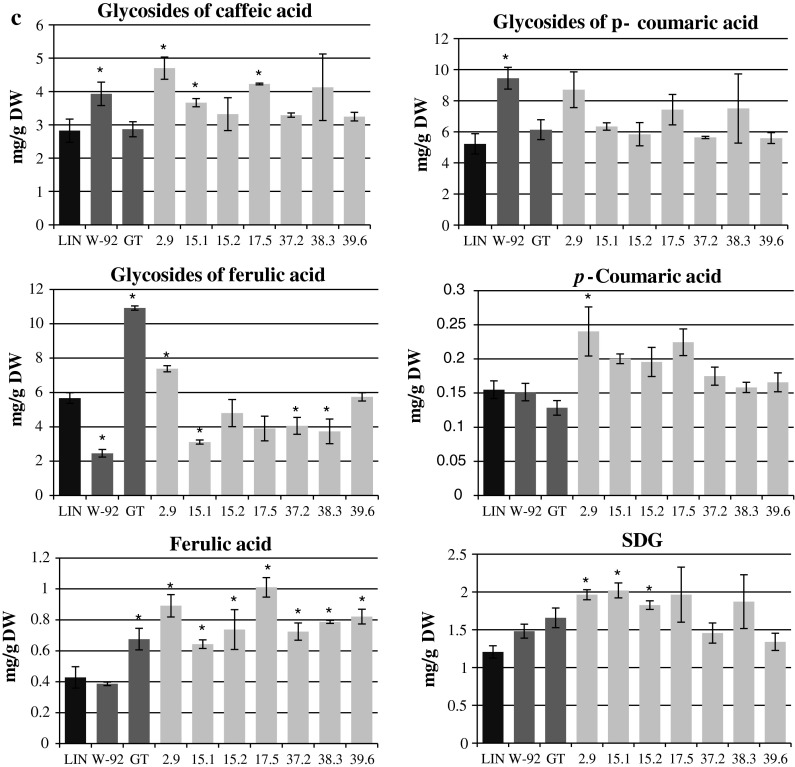
**a** The contents of free (unbound) caffeic acid derivative and free flavonoids identified in methanol extracts from stems of the W92 × GT, control (LIN), maternal (W92) and paternal (GT) plants. The contents of the compounds were calculated based on the areas of the peaks in relation to standard compounds of known concentration. The calculations were performed using Empower software. The results are the mean values ± SD of three independent experiments (*n* = 3). *Statistically significant difference compared to LIN at *p* < 0.05. **b** The contents of cell-wall-bound phenylpropanoid compounds identified in the extracts after alkaline hydrolysis of the stems of W92 × GT, control (LIN), maternal (W92) and paternal (GT) plants. The contents of the compounds were calculated based on the peak areas in relation to standard compounds of known concentration using Empower software. The results are the mean values ± SD of three independent experiments (*n* = 3). *Statistically significant difference compared to LIN at *p* < 0.05. **c** The contents of secoisolariciresinol diglucoside (SDG) and other phenylpropanoid compounds identified in the seeds of W92 × GT, control (LIN), maternal (W92) and paternal (GT) plants, expressed as mg/g of non-defatted seeds. The contents of the compounds were calculated based on the peak areas in relation to standard compounds of known concentration using Empower software. The results are the mean values ± SD of three independent experiments (*n* = 3). *Statistically significant difference compared to LIN at *p* < 0.05. *DW* dry weight

In the stems of the studied plants, the main free (unbound) phenylpropanoids were identified as isoorientin (luteolin 6-C-glucoside), isovitexin (apigenin-6-C-glucoside), vitexin and a caffeic acid derivative (containing at least two molecules of caffeic acid based on the MS spectrum) (Fig. 2a). The mean contents of the flavonoids assayed in all of the W92 × GT lines were higher than in the control and parental plants.

All of the lines showed higher isoorientin, isovitexin and vitexin contents than the control and maternal plants. Statistically significant differences were observed for lines 17.5, 38.3 and 39.6. Relative to the values for the paternal plants, isoorientin content was higher in five W92 × GT lines, isovitexin in two lines and vitexin in four lines. The highest contents of the flavonoids assayed were found in lines 15.2 and 38.3, which respectively had 244 and 289 % more isoorientin than the control, 150 and 183 % more than the maternal plants, and 118 and 146 % more than the paternal plants.

The caffeic acid derivative content was higher than in the control plants, but similar to that for the parental plants. Line 38.3 had a distinctive content of caffeic acid derivative: 263 % higher than the control plants, 103 % higher than the maternal plants, and 149 % higher than the paternal plants.

The content of phenolic compounds that are ester-bound to the cell wall was determined and is presented in Fig. 2b. Vanilic acid, vanillin, *p*-coumaric acid, syringic aldehyde, ferulic acid, 4-hydroxybenzoic aldehyde and vitexin were identified as the main components. The vanilic acid content in the stems of the plants studied was higher than that in the control plants (statistically significant differences were observed in all of the lines except 17.5 and 38.3), although none of the W92 × GT plants showed significant vanilic acid contents in comparison to the parental plants. Three lines (15.1, 37.2 and 39.6) showed higher vanillin contents relative to the control plants. None of the W92 × GT crossbreeds showed significantly higher vanillin content than the parental plants.

The *p*-coumaric acid contents in the crossbred plants were lower than in the control, and similar to the content in the parental plants. Only line 2.9 was different, with a 70 % higher *p*-coumaric acid content than the maternal plants, a 132 % higher content than the paternal plants and a 38 % higher content than the control plants.

The majority of the lines showed a higher content of ferulic acid. Lines 2.9, 15.1, 37.2 and 39.6 had significantly higher contents of syringic aldehyde than the control. Significantly higher amounts of syringic aldehyde were found in line 2.9: 74 % higher than the maternal plants, 22 % higher than the paternal plants, and 125 % higher than the control plants. None of the lines showed a significantly higher content of 4-hydroxybenzoic acid or vitexin bound to the cell wall.

During the chromatographic separation of the flax stem extracts, other presumed phenylpropanoid compounds were found, but could not be identified unambiguously. The contents of those free, unidentified phenylpropanoid compounds in W92 × GT flax were higher than in the control and parental plants (Supplementary Table S1). The contents of the cell-wall-bound compounds were similar to or slightly lower than in the control and parental plants (Supplementary Table S1).

### Levels of secoisolariciresinol diglucoside (SDG) and other phenylpropanoid compounds in flax seeds

After assessing the phenylpropanoid compound contents in the stems of the W92 × GT plants, we analyzed the content of these compounds in the seeds. The most attention was paid to SDG, as an increase in its content was noted in the parental plants in previous studies (Czemplik et al. 2011; Lorenc-Kukuła et al. 2009). SDG is formed as a result of the combination of two molecules of coniferyl alcohol. This compound enters the lignan complex comprising SDG, ferulic, coumaric and caffeic acid glucosides (Kosińska 2011; Meagher et al. 1999) and, as reported in some publications, herbacetin diglucoside (Struijs et al. 2007) cross-linked with 3-hydroxy-3-methylglutaric acid.

The W92 × GT lines 2.9, 15.1, 15.2, 17.5, 37.2 and 38.3 contained 63, 67, 51, 63, 21 % and 55 % more SDG, respectively, than the control plants (LIN), but statistically significant differences were only observed in the cases of lines 2.9, 15.1 and 15.2. Lines 2.9, 15.1, 15.2, 17.5 showed 33, 36, 23 and 33 % higher SDG content, respectively, than the maternal plants, and, again, statistically significant differences were only observed in lines 2.9, 15.1 and 15.2. The SDG levels were 18, 22, 10 and 19 % higher, respectively, than those in the paternal plants, with the only statistically significant difference found in line 15.1. To summarize, the W92 × GT plants, similarly to their parental plants, contained higher amounts of SDG in comparison to the *Linola* flax variety. The data are presented in Fig. 2c.

The following compounds were also identified in W92 × GT flax seeds: caffeic acid glucoside, *p*-coumaric acid glucoside, ferulic acid glucoside, *p*-coumaric acid and ferulic acid. The content of caffeic acid in W92 × GT flax was higher than in the*Linola* control and paternal plants (statistically significant for lines 2.9, 15.1 and 17.5). Lines 2.9, 15.1 and 17.5 of the W92 × GT plants showed 67, 21 and 42 % higher contents of *p*-coumaric acid glucoside, respectively, compared to the control plants. Only line 2.9 had a higher content of caffeic acid glucoside than the maternal plants (20 % higher), a higher content of *p*-coumaric acid glucoside than the paternal plants (42 % higher), and a higher content of ferulic acid glucoside than the control plants (30 % higher). None of the W92 × GT lines had a higher *p*-coumaric acid glucoside content than the maternal plants or a higher ferulic acid glucoside content than the paternal plants. All of the W92 × GT lines had higher contents of ferulic acid glucoside than the maternal plants. The data are presented in Fig. 2c.

Lines 2.9, 15.1, 15.2 and 17.5 showed higher contents of *p*-coumaric acid than the control and maternal plants, although the only statistically significant difference was in line 2.9. All the lines studied had higher contents of this compound than the paternal plants. The highest content of *p*-coumaric acid was found in line 2.9 and it was 55 % more than in the control plants, 59 % more than in the maternal plants, and 87 % more than in the paternal plants.

All the W92 × GT lines studied had higher contents of ferulic acid than the control and maternal plants. The lowest amount of this compound was found in line 15.1, but it was still 50 % higher than in the control plants and 66 % higher than in the maternal plants. The highest content of this compound was found in lines 2.9 and 17.5: respectively 108 and 136 % higher than in the control, 131 and 162 % higher than in the maternal plants, and 32 and 50 % higher than in the paternal plants. The W92 × GT crossbreeds had similar ferulic acid contents to the paternal plants. The data are presented in Fig. 2c.

### Fatty acid contents in flax seeds

The parental plants were derived from the oil variety of flax (*Linola*). Its seeds accumulate oil with significantly higher α-linoleic acid content than the traditional varieties. The W92 × GT flax line had a similar content of this acid to the original line (LIN) (Table 1). Crossbreeding the transgenic lines did not significantly affect the fatty acid content in the seeds of the progeny plants. Slight differences between the oleic acid (C18:1) and linoleic acid (C18:2) contents can be observed in the plants studied (Table 1). The differences lie between the values for the parental plants.

**Table 1:** Fatty acid composition in crossbred lines (numbered) in comparison to the parental W92 and GT lines and the control (Lin)

Fatty acid	Plant line									
	Lin	W92	GT	2.9	15.1	15.2	17.5	37.2	38.3	39.6
C16:0	8.7 ± 0.53	6.7 ± 0.66	6.9 ± 0.39*	7.2 ± 0.63*	7.7 ± 0.15	6.6 ± 0.49*	7.8 ± 0.16	8.4 ± 0.46	7.5 ± 0.3	7.7 ± 0.47
C18:0	2.9 ± 0.24	3.5 ± 0.1	3.1 ± 0.53	2.3 ± 0.04	2.6 ± 0.09	3.5 ± 0.22	2.9 ± 0.16	3.3 ± 0.23	3.3 ± 0.36	3.5 ± 0.39
C18:1	15.9 ± 1.7	13.9 ± 0.03	37.3 ± 0.64*	19.7 ± 2.53	20.1 ± 1.00	29.2 ± 2.69*	18.1 ± 1.42	23.1 ± 4.18	22.5 ± 2.99	24.4 ± 3.04
C18:2	70.1 ± 1.00	74.2 ± 0.61	51.6 ± 0.67*	69.1 ± 1.36	67.6 ± 0.82	59.4 ± 3.18*	69.8 ± 1.65	63.9 ± 4.83	65.5 ± 2.92	62.9 ± 3.91
C18:3	2.4 ± 0.77	1.7 ± 0.13	1.2 ± 0.15	1.9 ± 0.6	2 ± 0.22	1.3 ± 0.23	1.4 ± 0.07	1.3 ± 0.01	1.2 ± 0.09	1.5 ± 0.06

* Statistically significant difference compared to control at *p* < 0.05

### Antioxidative properties

**Fig. 3:**
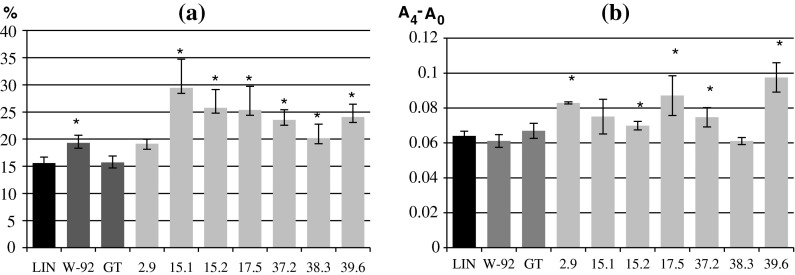
**a** Analysis of antioxidative properties of extracts from W92 × GT, control (LIN) and parental (W92, GT) plants measured with the DPPH method. The results are presented as the percentage of inhibition of free-radical reaction as described in the “Materials and methods” section. The results are the means of three repetitions; the bars represent standard deviations. *Statistically significant difference compared to LIN at *p* < 0.05. **b** Analysis of antioxidative properties of extracts from W92 × GT, control (LIN) and parental (W92, GT) plants measured with the FRAP method according to the description in the “Materials and methods” section. The results are the means of three repetitions; the bars represent standard deviations. *Statistically significant difference compared to LIN at *p* < 0.05. *DW* dry weight. **a** Antioxidant potential-DPPH, **b** antioxidant potential-FRAP

The antioxidative properties of the W92 × GT plants examined were assayed using the DPPH method and the results are presented in Fig. 3. The paternal plants showed similar antioxidative properties to the control plants, while the maternal plants had higher antioxidative potential. All of the W92 × GT lines except 2.9 had significantly higher antioxidative potentials than the control.

To confirm the DPPH results, the antioxidative properties of the plants studied were assayed using the FRAP method. The paternal plants’ antioxidative properties were similar to those obtained for the control plants. None of the W92 × GT plants examined showed weaker antioxidative properties than the control and parental plants. Lines 2.9, 15.2, 17.5, 37.2 and 39.6 plants showed better antioxidative properties, and the differences were statistically significant.

The majority of the W92 × GT lines examined showed better antioxidative properties when assessed with either method. Line 2.9 showed significantly higher antioxidative potential than the control plants when assessed using the FRAP method, but did not show better antioxidative properties when the DPPH method was used. Lines 15.1 and 38.3 demonstrated better antioxidative properties when assessed with the DPPH method, but this was not confirmed by the FRAP results.

### Assessment of tissue culture plant resistance to *F. culmorum* and *F. oxysporum*

The main pathogens of flax are fungi of the *Fusarium* family. It was expected that the increase in the levels of antioxidative compounds, including glucoside derivatives of flavonoids, in the transgenic plants would raise their resistance to infection. The results confirmed that W92 × GT plants showed better resistance to *F. culmorum* and *F. oxysporum* than the control and parental plants.

Non-transgenic plants attacked by *F. culmorum* became yellowish and their leaves wilted, while the W92 × GT plants remained green. Moreover, the paternal plants shower higher resistance to the pathogen than the maternal plants.

**Fig. 4:**
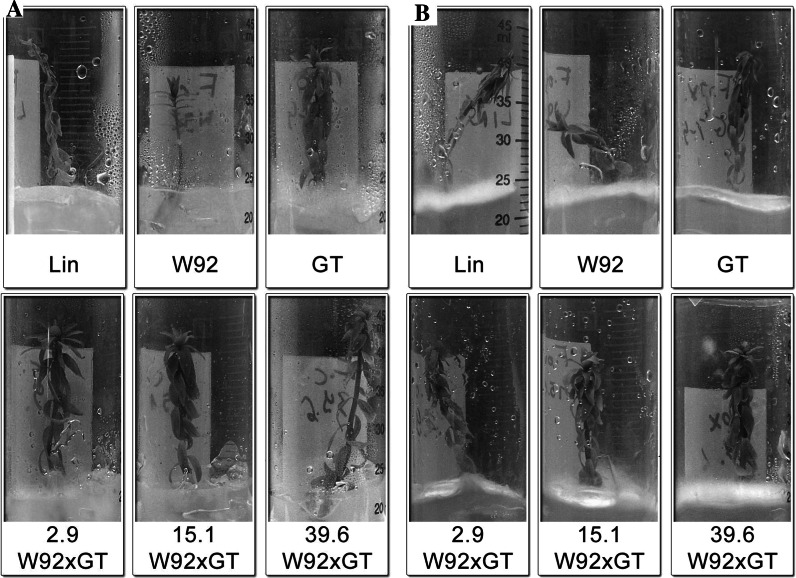
**a** Examples of W92 × GT, control (LIN) and parental (W92, GT) plants after infection with *F. culmorum* in tissue culture. **b** Examples of W92 × GT, control (LIN) and parental (W92, GT) plants after infection with *F. oxysporum* in tissue culture

The control plants infected with *F. oxysporum* turned brownish-yellow, their leaves became coiled, and the stem bent. The stems of the maternal plants also bent, and their leaves became yellowish, though not to the same extent as those of the control plants. No significant bend was observed in the paternal plant stems, and their leaves were greener and only slightly coiled. The W92 × GT plants had still greener leaves and their stems remained unbent. Figure 4 shows examples of transgenic and control plants infected with *F. culmorum* and *F. oxysporum*.

## Discussion

Flax is a crop that is used in many branches of industry. It is a source of oil that is rich in polyunsaturated fatty acids and its fibers contain antioxidants. Poland has a long-standing tradition of flax cultivation, but in the past few decades the level of cultivation has decreased due to a lack of new, beneficial varieties. Several attempts to generate flax with improved properties have been made in recent times.

Flavonoids are a group of secondary plant metabolites with diverse chemical structures. They are very active natural antioxidants that play many important roles in a plant, including acting as antimicrobials and protecting the plant from various stress factors. They can be deterrents against herbivores and they occur as pigments in fruits, flowers and seeds.

Our goal was to generate a flax variety with a higher flavonoid content that would be beneficial for the medical, dietary and/or industrial applications of the plant components. One method applied involves raising the flavonoid content by overexpressing a gene or multiple genes engaged in the flavonoid biosynthesis pathway. Modifying CHI, one of the enzymes of the flavonoid synthesis pathway in tomato plants, led to a flavonoid content increase (Verhoeyen et al. 2002). In the case of potato plants, manipulating the diflavonol reductase gene also led to an increase in the flavonoid content (Łukaszewicz et al. 2004). In flax, three genes coding for the enzymes of the phenylpropanoid synthesis pathway were overexpressed: CHS, CHI and DFR. This also gave rise to an increase in the phenolic compound content (Lorenc-Kukuła et al. 2005).

Another way to increase the flavonoid pool is to improve the compounds’ stability, for example by overexpressing the genes encoding GTs, which are responsible for the glucosylation process of phenylpropanoid compounds. In vitro studies (Ross et al. 2001) and earlier research on flax (Lorenc-Kukuła et al. 2009) showed that glycosylation of flavonoids led to a decrease in their activity, thus increasing their stability.

Two methods are employed to obtain new varieties of plants: crossbreeding and transgenesis. In this study, the methods were combined. The plants that we generated did not show significant phenotypic differences from the non-modified plants.

Earlier experiments on transgenic potatoes with elevated flavonoid levels showed decreased yields (Łukaszewicz et al. 2002), but this was not observed in the plants in this study, nor in earlier studies on W92 and GT flax. Three flavonoids were identified in the stems of the plants investigated: isoorientin, isovitexin and vitexin. The contents of the identified flavonoids were higher than in the control and parental plants, which concurs with those earlier results (Lorenc-Kukuła et al. 2005, 2009). The amounts of the remaining free phenylpropanoid compounds in the stems were also higher than in the control and parental plants.

A significant increase in SDG content in the seeds of the parental plants was noted in earlier studies (Czemplik et al. 2011; Lorenc-Kukuła et al. 2009). The W92 × GT plants, similar to their parental plants, had higher amounts of SDG than the *Linola* flax variety (LIN). Those studies showed that the phenolic acid content increased in the seeds of W92 (Lorenc-Kukuła et al. 2005; Żuk et al. 2011) and GT plants (Lorenc-Kukuła et al. 2009).

In the seeds of the W92 × GT plants, phenolic acids and their glucosides were assayed, and their levels were found to be similar to those in the parental plants. Crossbreeding the transgenic plants did not significantly influence the content of the fatty acids assayed in the seeds of the progeny plants. Slight differences were found between the contents of oleic acid (C18:1) and linoleic acid (18:2) in the plants investigated, and these lay between the values for the parental plants.

One of the aims of this study was to enrich flax in natural antioxidants by increasing the content and stability of flavonoids, which have strong antioxidative properties. Because of the diversity of natural antioxidants in plants, more than one method to assay their antioxidative potential is advised. In this study, we used both the DPPH and FRAP methods. The DPPH method exploits the ability of antioxidants to quench free radicals (Williams et al. 1995), while the FRAP method is based on the antioxidant-driven reduction of Fe(III)–Fe(II) ions (Benzie and Strain 1996).

The majority of the W92 × GT lines examined showed better antioxidative properties with both assessment methods. The slight differences in the results obtained may be due to the different mechanisms involved in these methods. The FRAP method encompasses more antioxidants in the sample, while the DPPH method detects only the most reactive portion.

Flavonoids and other phenylpropanoids play an important role in the protection of flax against pathogen infection (Kostyn et al. 2012), so studies on looking at infection of the crossbreeds with *F. culmorum* and *F. oxysporum* could not be omitted. These fungi are the main pathogens of flax. In previous studies, the maternal plants (W92) proved to be about 40 % more resistant to *F. culmorum* and *F. oxysporum* than the control plants (Lorenc-Kukula et al. 2005). The resistance of the paternal plants to *F. culmorum* infection was about 70 % higher than that of the control plants, and in the case of *F. oxysporum* about 90 % higher (Lorenc-Kukula et al. 2009). Those earlier studies considered the number of infected seedlings.

A different approach was taken in this study. The influence of the pathogens on 4-week-old plants from tissue culture was assessed. Due to the difference in the investigation method, the degree of increased resistance of W92 and GT plants found in this and previous studies cannot be unequivocally compared. However, it is noticeable that in this and in those previous studies, the paternal plants showed higher resistance to the infection than the maternal plants. The investigated W92 × GT crossbreeds showed even better resistance to fungal infection than the parental plants. It is clear that crossbreeding the transformants that overproduce flavonoids with the transformants that overexpress the gene responsible for the glucosylation of flavonoids positively influences the resistance of the resultant flax to fungal infections.

We investigated the influence of transgenic plants on the properties of the progeny plants. In this case, crossbreeding positively influenced the properties of the generated plants. They showed very similar or, in many cases, more beneficial features than the parental plants and the non-modified plants of the initial variety.

In all of our experiments, differences between the individual W92 × GT plant lines were visible. This can be due to the different levels of expression of all the exogenous genes, resulting from the random integration of the gene construct into the plant genome. The differences may also be due to the different numbers of integrated transgene copies (Gadaleta et al. 2011). Therefore, it might prove relevant to conduct an investigation to determine the number of copies integrated with the genome. Studies on the inheritance of the genes overexpressed in the parental plants would be also helpful. Another important aspect is the stability of the crossbreeds obtained, so it would be important to investigate whether the features obtained are passed on to the next generations. Studies have been done on obtaining crossbreeds by crossing transgenic wheat (Li et al. 2007) and rice (Wang et al. 2004). However, no data on crossbreeds of transgenic flax plants have been published. Our study indicates that this is possible. If further investigations demonstrate the stability of such crossbreeds, it will generate a new possibility of obtaining flax with improved applicable attributes.

## Electronic supplementary material

Below is the link to the electronic supplementary material.

**Supplementary Figure S1.** Electrophoretic separation of PCR products amplified with primers to detect the presence of the 5UGT gene (panels **a** and **c**) and DFR gene (panels **b** and **d**) on a DNA matrix isolated from the green parts of W92 × GT flax plants (leaves and stems) from the F1 (panels **a** and **b**) and F2 generations (panel **c** and **d**). **Lin** – negative control (control plant); **Pl** – positive control: plasmid containing the 5UGT gene (panels **a** and **c**) or DFR gene (panels **b** and **d**); **W92** – transgenic flax with overexpression of the DFR gene (maternal plant); **GT** – transgenic flax with overexpression of the 5UGT gene (paternal plant); **C** – reagent purity control. The individual lines generated as the result of the crossbreeding are numbered (DOCX 431 kb)

**Supplementary Figure S2.** Analysis of lignins, pectins and cellulose in the cell walls of dried stems of W92 × GT, control (LIN) and parental (W92, GT) plants as described in the Materials and Methods section. The results are the means of three repetitions, the bars represent standard deviations, * – statistically significant result compared to LIN, at p < 0.05 (DOCX 19 kb)

**Supplementary Table S1** The analysis of free phenylpropanoid compounds that could not be identified unambiguously in the methanol extracts from the stems of W92 × GT, control (LIN), maternal (W92) and paternal (GT) plants. Because of the lack of the standard compounds, the data is presented in relation to the levels found in the LIN plants. Presumable identification of the compounds and their retention times and absorption maxima are presented in the table. The contents of the compounds were calculated based on the peak areas using Empower software. The results are the mean values of three independent experiments (n = 3 ± SD, p < 0.05). (DOC 35 kb)

**Supplementary Table S2** The content of cell wall-bound phenylpropanoid compounds that could not be identified unambiguously in the methanol extracts after alkaline hydrolysis of the stems of W92 × GT, control (LIN), maternal (W92) and paternal (GT) plants. Because of the lack of the standards compounds, the data is presented in relation to the levels found in the LIN plants. Presumable identification of the compounds and their retention times and absorption maxima are presented in the table. The contents of the compounds were calculated based on the peak areas using Empower software. The results are the mean values of three independent experiments (n = 3 ± SD, p < 0.05) (DOC 29 kb)
