# Supplementary figures and images for: Crossbreeding of transgenic flax plants overproducing flavonoids and glucosyltransferase results in progeny with improved antifungal and antioxidative properties
Source: Mol Breed. 2014 Aug 21;34(4):1917–32. doi: 10.1007/s11032-014-0149-5 (PMC4257994; doi:10.1007/s11032-014-0149-5)

**Fig. S1**

a
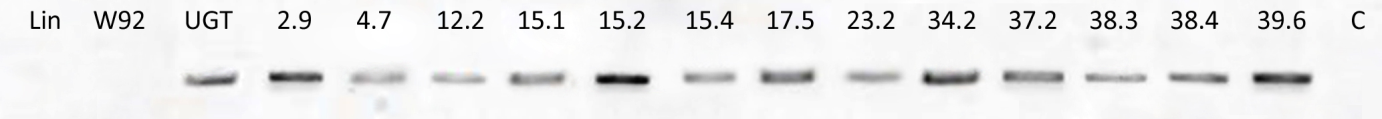
b
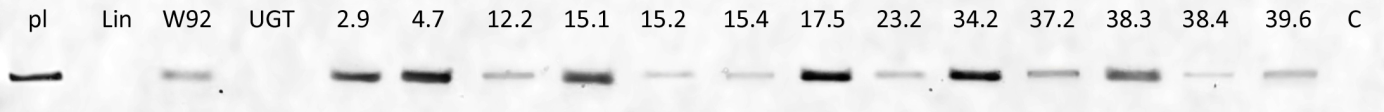
c
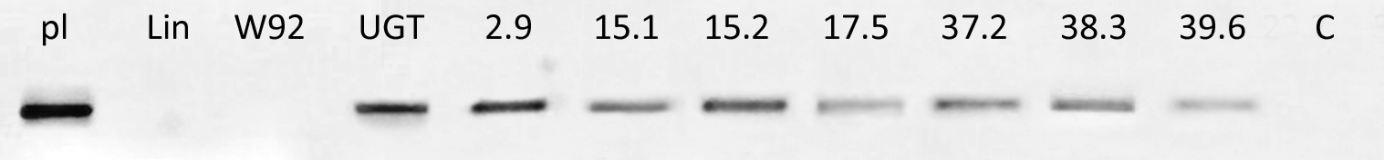
d
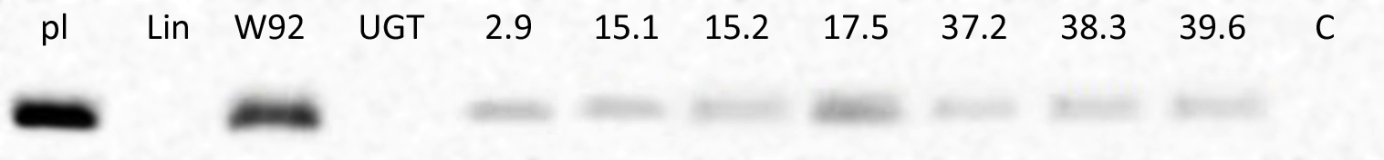

Supplement: Supplementary file 1 — Supplementary Figure S1. Electrophoretic separation of PCR products amplified with primers to detect the presence of the 5UGT gene (panels a and c) and DFR gene (panels b and d) on a DNA matrix isolated from the green parts of W92 × GT flax plants (leaves and stems) from the F1 (panels a and b) and F2 generations (panel c and d). Lin – negative control (control plant); Pl – positive control: plasmid containing the 5UGT gene (panels a and c) or DFR gene (panels b and d); W92 – transgenic flax with overexpression of the DFR gene (maternal plant); GT – transgenic flax with overexpression of the 5UGT gene (paternal plant); C – reagent purity control. The individual lines generated as the result of the crossbreeding are numbered (DOCX 431 kb) [file 11032_2014_149_MOESM1_ESM.docx]
