# Supplementary material for: Crossbreeding of transgenic flax plants overproducing flavonoids and glucosyltransferase results in progeny with improved antifungal and antioxidative properties
Source: Mol Breed. 2014 Aug 21;34(4):1917–32. doi: 10.1007/s11032-014-0149-5 (PMC4257994; doi:10.1007/s11032-014-0149-5)
Supplement: Supplementary file 2 — Supplementary Figure S2. Analysis of lignins, pectins and cellulose in the cell walls of dried stems of W92 × GT, control (LIN) and parental (W92, GT) plants as described in the Materials and Methods section. The results are the means of three repetitions, the bars represent standard deviations, * – statistically significant result compared to LIN, at p < 0.05 (DOCX 19 kb) [file 11032_2014_149_MOESM2_ESM.docx]

**Supplementary Figure S2**
