# Supplementary material for: Crossbreeding of transgenic flax plants overproducing flavonoids and glucosyltransferase results in progeny with improved antifungal and antioxidative properties
Source: Mol Breed. 2014 Aug 21;34(4):1917–32. doi: 10.1007/s11032-014-0149-5 (PMC4257994; doi:10.1007/s11032-014-0149-5)
Supplement: Supplementary file 3 — Supplementary Table S1 The analysis of free phenylpropanoid compounds that could not be identified unambiguously in the methanol extracts from the stems of W92 × GT, control (LIN), maternal (W92) and paternal (GT) plants. Because of the lack of the standard compounds, the data is presented in relation to the levels found in the LIN plants. Presumable identification of the compounds and their retention times and absorption maxima are presented in the table. The contents of the compounds were calculated based on the peak areas using Empower software. The results are the mean values of three independent experiments (n = 3 ± SD, p < 0.05). (DOC 35 kb) [file 11032_2014_149_MOESM3_ESM.doc]

**Supplementary Table S1**

| IP | RT | Maximum of absorbance | **LIN=1** | | | | | | | | |
| --- | --- | --- | --- | --- | --- | --- | --- | --- | --- | --- | --- |
|  |  |  | **W92** | **UGT** | **2.9** | **15.1** | **15.2** | **17.5** | **37.2** | **38.3** | **39.6** |
| **Unidentified**  **Presumably a form of chlorogenic acid** | 3,956 | 225.5; 324.7 | 1,31 ±0,03 | | 1,00 ±0,05 | | --- | | | 1,58 ±0,02 | | --- | | | 1,71 ±0,33 | | --- | | | 1,91 ±0,27 | | --- | | | 1,96 ±0,19 | | --- | | | 1,95 ±0,26 | | --- | | | 2,89 ±0,51 | | --- | | | 2,16 ±0,01 | | --- | |
| **Unidentified**  **Presumably a form of chlorogenic acid** | 4,386 | 223.7; 326.5 | **1,14 ±0,11**   |  | | --- | | **1,01 ±0,11** | | 1,49 ±0,10 | | --- | | | 1,25  ±0,14 | | --- | | | 1,15 ±0,04 | | --- | | **1,03 ±0,06** | | 1,018 ±0,11 | | --- | | 1,53 ±0,13 | 1,38 ±0,09 |
| **Unidentified**  **Presumably  apigenin di-C-glucoside** | 5,620 | 227.9; 271.8; 339.5 | 1,05 ±0,01 | 0,86 ±0,11 | 0,90 ±0,10 | 1,01 ±0,18 | 1,31 ±0,20 | 1,22 ±0,12 | 1,12 ±0,12 | 1,58 ±0,13 | 1,19 ±0,01 |
| **Unidentified**  **Presumably a form of ferulic acid** | 8,348 | 227.3; 274.9; 328.4 | 1,28 ±0,07 | 1,23 ±0,12 | 1,17 ±0,14 | 1,53 ±0,23 | 2,25 ±0,46 | 1,94 ±0,19 | 1,58 ±0,10 | 2,81 ±0,12 | 2,11 ±0,21 |
